# Rare Norovirus GIV Foodborne Outbreak, Wisconsin, USA

**DOI:** 10.3201/eid2704.204521

**Published:** 2021-04

**Authors:** Leslie Barclay, Tim Davis, Jan Vinjé

**Affiliations:** Centers for Disease Control and Prevention, Atlanta, Georgia, USA (L. Barclay, J. Vinjé);; Wisconsin State Laboratory of Hygiene, Madison, Wisconsin, USA (T. Davis)

**Keywords:** norovirus, viruses, genotype, GIV, foodborne diseases, foodborne outbreak, food safety, fruit, Wisconsin, United States

## Abstract

We report a norovirus GIV outbreak in the United States, 15 years after the last reported outbreak. During May 2016 in Wisconsin, 53 persons, including 4 food handlers, reported being ill. The outbreak was linked to individually prepared fruit consumed as a fruit salad. The virus was phylogenetically classified as a novel GIV genotype.

Norovirus is the leading cause of epidemic and endemic acute gastroenteritis globally. The virus can be transmitted through person-to-person contact, consumption of fecally contaminated food or water, or self-contamination after touching contaminated environmental surfaces (*1*,*2*). Noroviruses are divided into at least 10 genogroups (G), and viruses in GI, GII, GIV, GVIII, and GIX cause illness in humans (*3*). More than 99% of all norovirus outbreaks are caused by GI and GII viruses in the United States (*4*). GVIII includes 2 strains that have been detected in Japan during 2004 and 2011 (*3*), and GIX has caused 11 reported outbreaks in the United States since 2013 (https://www.cdc.gov/norovirus/reporting/calicinet/data.html).

GIV is divided into 2 recognized genotypes: GIV.1, which infects humans (*5*), and GIV.2, which infects canines and felines (*6*). GIV viruses were reported in humans in the Netherlands during 1998 and the United States during 1999 (*7*,*8*) and have since been sporadically reported in clinical and environmental samples (*5*,*9*–*11*). An outbreak linked to a GIV norovirus in the United States has not been reported since 2001 (*4*,*8*). In this article, we describe a 2016 foodborne norovirus outbreak associated with a novel GIV strain (tentatively GIV.NA).

## The Study

**Figure 1:**
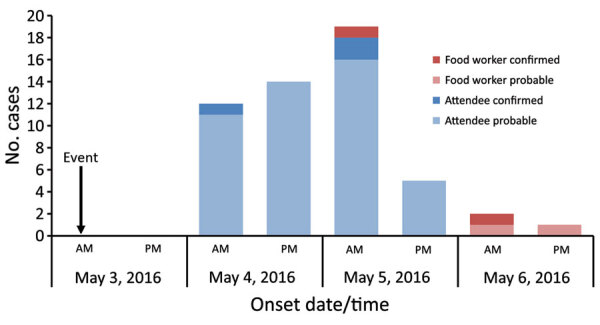
Epidemiologic curve of attendees and food handlers by date of illness onset for event during rare norovirus GIV foodborne outbreak, Wisconsin, USA. Arrow indicates event date and time period event occurred. am indicates 12:00 am–11:59 am and pm indicates 12:00 pm–11:59 pm. Dark red indicates laboratory-confirmed food workers, light red indicates probable food workers, dark blue indicates laboratory-confirmed attendees, and light blue indicates probable attendees.

On May 6, 2016, the Wisconsin Department of Public Health was notified of a possible norovirus outbreak. The outbreak occurred at a breakfast event held at a restaurant on May 3, 2016, for local business owners (Figure 1). According to interviews of the affected group, 49 attendees and 4 food handlers reported being ill, and the first case was reported on May 4, 2016. The peak of illness occurred 48 hours after the breakfast event. Symptoms included diarrhea, vomiting, and nausea (Table 1). Duration of illness was 1–5 days (median 2 days), and incubation time range was 15–57 hours (median 38 hours).

**Table 1:** Clinical and demographic characteristics of attendees associated with a norovirus GIV foodborne outbreak, Wisconsin, USA*

Characteristic	Value
Onset of illness	2016 May 4
Total no. ill	49
Hospitalized	0
Died	0
Duration of illness, d	2
Primary case-patients	49
Laboratory confirmed	3
Probable	46
Secondary case-patients	0
Laboratory confirmed	0
Probable	0
Sex	
M	13 (26.5)
F	34 (69.4)
Unknown	2 (4.1)
Age range, y (median)	17–77 (47)
Age group, y	
<1	0 (0)
1–4	0 (0)
5–9	0 (0)
10–19	2 (4.1)
20–49	24 (49.0)
50–74	14 (28.6)
>75	1 (2.0)
Unknown	8 (16.3)
Case-patients with known illness duration	35
Duration range, d	1–5
Duration median, d	2
Case-patients with known incubation period	49
Incubation range, h	15–57
Incubation median, h	38
Symptom	
Diarrhea	44 (89.8)
Nausea	41 (83.7)
Fatigue	41 (83.7)
Headache	35 (71.4)
Abdominal pain	34 (69.4)
Chills	31 (63.3)
Vomiting	30 (61.2)
Body ache	30 (61.2)

*Values are no. or no. (%) except as indicated. Clinical and demographic characteristics for the 4 food handlers associated with this outbreak are not available.

On the basis of the epidemiologic investigation, pathogen transmission occurred through foodborne exposure, and the highest risk ratio was linked to individually eaten fruit from a fruit salad served at the breakfast (risk ratio 2.17–3.29) (Table 2). The epidemiologic curve and risk ratios were calculated by using Microsoft Excel (https://www.microsoft.com) and R software (http://www.r-project.org). The food handlers prepared the fruit, which was served as a fruit salad during the meal on May 2, the day before the breakfast. Reportedly, the strawberries and grapes were washed, whereas the melons were not. There was no leftover fruit available for laboratory testing. 

**Table 2:** Analysis of implicated food and drink in a norovirus GIV outbreak, Wisconsin, USA*

Food and drink items implicated	Persons who ate food or drink			Persons who did not eat food or drink		RR (95% CI)	p value
	Total	% Ill		Total	% Ill		
Any quiche	74	58		5	60	0.95 (0.32–2.89)	0.934
Ham and cheese quiche	51	63		23	48	1.40 (0.83–2.38)	0.229
Vegetable quiche	21	48		54	63	0.71 (0.41–1.21)	0.226
Any fruit	71	65		7	0	2.84 (2.07–3.89)	<0.05
Cantaloupe	55	67		12	25	2.29 (1.39–3.78)	<0.05
Honeydew melon	52	65		12	25	2.17 (1.32–3.56)	<0.05
Grapes	58	69		9	0	3.22 (2.20–4.73)	<0.05
Strawberries	56	70		10	0	3.29 (2.22–4.90)	<0.05
Potato pancakes	63	63		16	38	1.71 (1.04–2.82)	0.060
Applesauce	41	68		35	46	1.71 (1.00–2.95)	<0.05
Muffins	50	62		25	52	1.26 (0.74–2.17)	0.407
Butter	9	78		64	56	1.97 (0.56–6.90)	0.219
Orange juice	60	63		17	47	1.44 (0.83–2.52)	0.227
Coffee	58	60		20	55	1.13 (0.64–2.03)	0.675
Creamer	21	67		53	55	1.36 (0.69–2.66)	0.348
Water	67	61		7	43	1.47 (0.73–3.00)	0.347

*RR, relative risk.

Nucleic acid was extracted from stool samples collected from 6 ill persons and tested for norovirus GI/GII by real-time quantitative reverse transcription PCR (qRT-PCR), and 5 GII-positive samples (from 3 attendees and 2 food handlers) with high cycle threshold values (range 28–37) were amplified by conventional RT-PCR targeting a partial region of the 5′ end of open reading frame 2 (*4*). The sequences did not cluster with any GI or GII norovirus reference sequences and closely matched GIV viruses (*4*).

**Figure 2:**
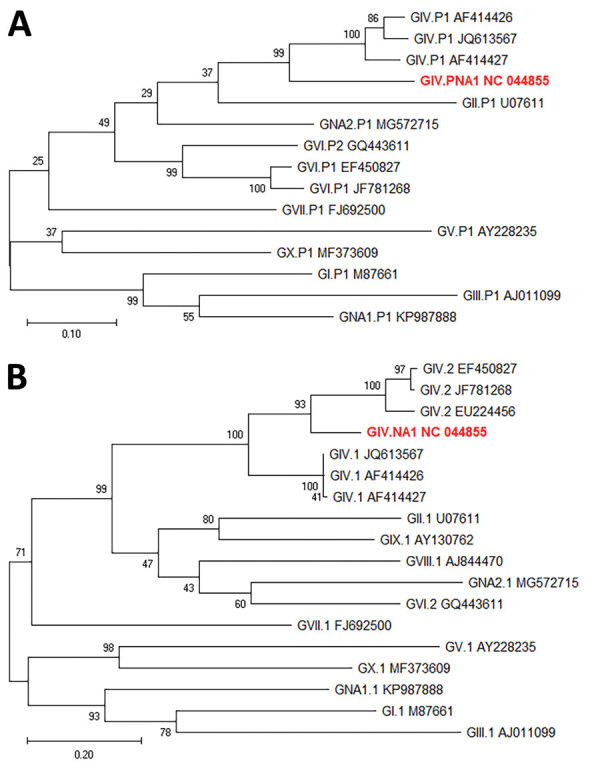
Maximum-likelihood phylogenetic analysis of rare norovirus GIV isolated during foodborne outbreak, Wisconsin, USA (red text), and reference strains. A) Partial polymerase gene (762 nt); B) complete capsid (VP1) gene (554 aa). Bootstrap support for 500 replicates is indicated on branches. For polymerase analysis, evolutionary distances were inferred by the Tamura-Nei model. For VP1 analysis, evolutionary distances were inferred by using the Jones-Taylor-Thornton matrix-based model. Reference strains are represented by type and GenBank accession number. Scale bar in panel A indicates nucleotide substitutions per site, and scale bar in panel B indicates amino acid substitutions per site.

Next-generation sequencing (NGS) was performed on 3 samples and verification of final consensus sequences was performed by using Geneious version 11.1.2 (Biomatters Inc., https://www.newjerseybids.us) (*4*). Sequences for all 3 near complete genomes (≈7,490 nt) were identical (GenBank accession no. NC_044855). The closest polymerase gene sequence in GenBank had a 79% nt similarity (Figure 2, panel A), and the capsid sequence matched partial capsid sequences derived from wastewater in Brazil, Japan, and the United States with a 98% nt identity. The closest complete major capsid sequence in GenBank (accession no. AF414426) had a 76% aa identity (Figure 2, panel B).

## Conclusions

We report a novel norovirus GIV genotype as the causative agent of a foodborne norovirus outbreak. Norovirus GIV outbreaks are rare and were reported in the Netherlands during 1998, the United States during 1999, and in Australia during ­­­­­2010 (*5*,*7*,*8*) and have since been detected sporadically in clinical samples (*9*–*11*). However, seroprevalence studies in Italy, the Netherlands, and the United States have shown that 19%–31% of these populations have antibodies against GIV (*12*–*14*). Possible explanations include that most laboratories do not test for norovirus GIV or most infections are asymptomatic or do not lead to a visit to a physician. However, 3 young children who were positive for GIV in a study in Italy had severe endemic acute gastroenteritis symptoms (*10*).

Several studies have detected norovirus GIV in rivers and wastewater (*9*–*11*,*15*); the number of positive samples ranged from 8.2% to 34%, further supporting that norovirus GIV is circulating in the general population. Several short sequences detected in wastewater collected in Brazil, Japan, and the United States match the capsid sequence in our study (*9*,*15*). However, new norovirus genotypes require >2 nonidentical complete capsid sequences from different geographic locations that form a separate phylogenetic cluster (*3*) Therefore, the virus detected in this outbreak cannot officially be assigned as GIV.3 yet but is assigned GIV.NA1[PNA1].

Detection of a norovirus GIV strain associated with a foodborne outbreak shows that despite the absence of reported GIV norovirus outbreaks over the past 15 years in the United States, these viruses continue to circulate in the human population. Because samples from endemic acute gastroenteritis outbreaks are typically tested only for noroviruses GI and GII, including testing of norovirus-negative samples for GIV might improve determining endemic acute gastroenteritis outbreaks of unknown etiology.
